# Disentangling the origins of viticulture in the western Mediterranean

**DOI:** 10.1038/s41598-023-44445-4

**Published:** 2023-10-12

**Authors:** Francesco Breglia, Laurent Bouby, Nathan Wales, Sarah Ivorra, Girolamo Fiorentino

**Affiliations:** 1 Department of Geosciences, University of Padua, 35131 Padua, Italy; 2 Laboratory of Archaeobotany and Palaeoecology, Cultural Heritage Department, University of Salento, 73100 Lecce, Italy; 3 Institut des Sciences de l’Evolution, University of Montpellier, 34095 Montpellier, France; 4 Department of Archaeology, University of York, York, YO1 7EP UK

**Keywords:** Agroecology, Agricultural genetics, PCR-based techniques, Bioinformatics

## Abstract

We present direct evidence of early grape domestication in southern Italy via a multidisciplinary study of pip assemblage from one site, shedding new light on the spread of viticulture in the western Mediterranean during the Bronze Age. This consist of 55 waterlogged pips from Grotta di Pertosa, a Middle Bronze Age settlement in the south of the Italian peninsula. Direct radiocarbon dating of pips was carried out, confirming the chronological consistency of the samples with their archaeological contexts (ca. 1450–1200 BCE). The extraordinary state of conservation of the sample allowed to perform geometric morphometric (GMM) and paleogenetic analyses (aDNA) at the same time. The combination of the two methods has irrefutably shown the presence of domestic grapevines, together with wild ones, in Southern Italy during the Middle/Late Bronze Age. The results converge towards an oriental origin of the domestic grapes, most likely arriving from the Aegean area through the Mycenaeans. A parent/offspring kinship was also recognised between a domestic/wild hybrid individual and a domestic clonal group. This data point out a little known aspect of the diffusion of the first viticulture in Italy, and therefore in the western Mediterranean, which involved the hybridization between imported domestic varieties with, likely local, wild vines.

## Introduction

In order to reconstruct the history of grape vines (*Vitis vinifera* L.) domestication, several studies have consistently shown that this process originated in Western Asia and Caucasus^1–11^, and it resulted into the differentiation of phenotypically distinct domestic (*Vitis vinifera* subsp. *vinifera*) and wild (*Vitis vinifera* subsp. *sylvestris*) subspecies. A recent study clarified that two separate domestication events took place concurrently around the advent of agriculture (~ 10.500 to 12.500 BP) in the two areas^12^. There is uncertainty however regarding what happened outside the primary area of domestication, especially concerning the role played by local wild populations present in those areas where viticulture developed over time. Numerous genetic studies have highlighted a complex relationship between the two subspecies, variously interpreted as the result of introgressive hybridisation following the introduction of domestic varieties from the eastern Mediterranean or secondary domestication of local wild vines^4,6,10,11,13,14^. In addition, the timing of grapevine domestication and of its early spread in the Mediterranean is poorly known. Observing the current geographical distribution of wild vines^15–17^, it is worth exploring the relationship between the two subspecies in the areas where viticulture spread.

Archaeobotany can potentially play a fundamental role in understanding the phenomenon as it allows us to have a direct look at the plant remains within past anthropic contexts that testify the process. Nevertheless, the spread and domestication of grapevine are not easy to read, mainly because recognition of the characteristics that enable a *Vitis vinifera* seed to be identified as belonging to the domestic or wild subspecies is not immediate. Over the years, various methods for achieving this aim have been developed, initially based on morphological observations and on the measurement and calculation of biometric indices on grape seeds^18–20^. These methods have however been found to suffer from various limitations. Their reliability depends on the method used, on the size and diversity of the reference collections and on the state of conservation of the archaeological remains^21^ and they can give contrasted results^22^. More elaborate methods were subsequently developed, based on the analysis of 2D images, on the statistical analysis of various morphometric features and comparison with modern reference collections^23–25^. A promising method for future research has been recently proposed for the identification of grape pips in terms of their variety, which envisages a complete 3D scanning of the geometry of the seeds and then analysis based on machine learning methods^26^. Geometric Morphometric (GMM) analysis of ventral and lateral outlines of the pips using the Elliptic Fourier Transform method allow the identification of modern cultivars and a powerful discrimination of wild and domesticated grape pips^24,27–29^. Paleogenetic analysis has also proved to be a powerful tool, even if strictly dependent on the taphonomic processes and the state of conservation. Up to now the best results, for both paleogenetics and morphometrics, come from the extraction and analysis from waterlogged material. This type of remains is not very common but some studies have been done, mainly regarding advanced steps in the history of viticulture in Mediterranean France^30,31^ but also other areas^29,32^, including Italy^33^.

For its geographic position, the Italian peninsula—especially its southern part—presumably played a major role in the spread of viticulture in the Mediterranean, and from here to western and central Europe.

**Figure 1:**
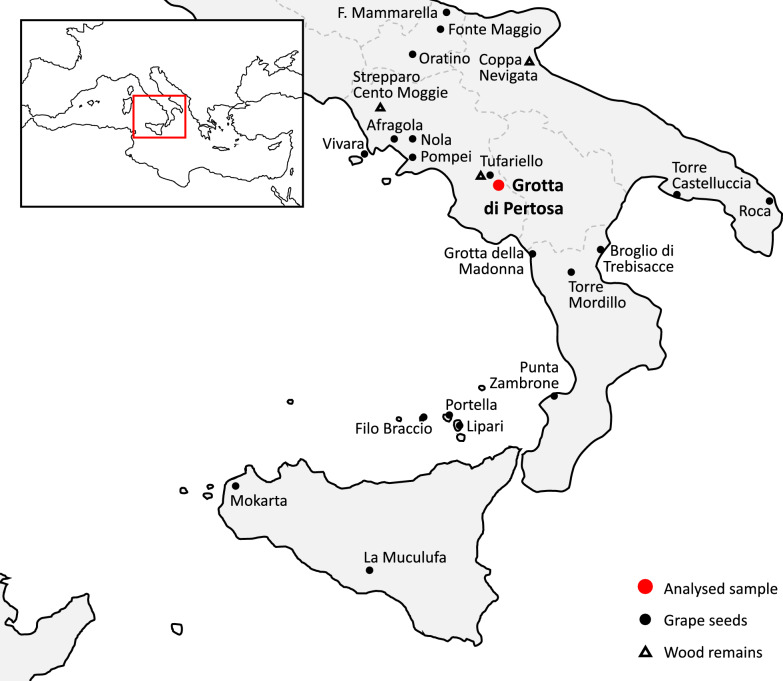
Bronze age sites with *Vitis vinifera* macro remains in southern Italy.

Previous studies of the assemblages of grape pips discovered in Italy by means of various biometric and morphometric methods do not always have the same degree of reliability and can yield results that are not always comparable with each other. However the general opinion is that during the Middle/Late Bronze Age the first evidence of seeds with domestic or hybrid characteristics began to become evident, mainly in northern Italy and Sardinia^34^. The use of non-comparable methods is not the only problem with the research on the diffusion of viticulture in this important geographical region. The lack of large grape pip assemblages from Southern Italy constitutes the main limit on our ability to read the phenomenon in detail and assess its implications for the morphology of grape pips (SI. Table S1). This paper is based on the study of one of the largest pips assemblages from protohistoric southern Italy, which chronology was ascertained by direct radiocarbon dating, and the only one made up of waterlogged specimens whose preservation state is suitable to simultaneously perform geometric morphometric and paleogenetic analysis: Grotte di Pertosa e Auletta hereinafter, for the sake of brevity, Grotta di Pertosa (Fig. 1).

### The site of Grotta di Pertosa and the context of discovery

The original layout of the settlement of Grotta di Pertosa seems to be dated to the early Middle Bronze Age. Its moment of greatest extension, which has been better investigated, was however in the immediately subsequent phase, which coincides with the Apennine facies and with the beginning of the Sub-Apennine facies in southern Italy, corresponding to the transition from the Middle to the Late Bronze Age^35,36^ (SI. Fig. S1). The site of Grotta di Pertosa lies in the hinterland of the Campania region in a strategic position dominating the northern entrance to the Vallo di Diano, a broad valley containing an ancient lake, now dried up, which forms a natural junction of several routes crossing the southern Apennines running both N-S and E-W. The decision to settle in the initial stretch of a large karst cave crossed by an underground river^37^ determined the type of construction. This consisted of pile-dwellings built mainly with the wood of deciduous oaks, partly constructed on the river’s muddy shore and partly directly in the water^35,38^ and it is the only known case of a pile-dwelling built inside a cave in the Mediterranean area. At least two overlapping levels of pile-dwellings have been recognized, but there were certainly a number of building phases^39–41^, considering that the settlement lasted until the early Iron Age^42,43^.

The silty-clay sediments in the cavity, saturated with water, have conserved the organic materials, and thus the plant remains, by waterlogging. However, for logistical reasons the site has not undergone systematic archaeological excavation in recent times. Despite this, during a survey in 2013, a well-conserved layer rich in plant remains was identified and interpreted as the floor of a collapsed pile-dwelling, from which a small sample (2 L of sediment) was taken^41^. The archaeobotanical analysis^35,38^ highlighted unequivocal traces of the processing of cereals and a clear preponderance of perennial plant fruits, which included the grape pips analysed in this study. In the sampled sediment 72 grape pips were found, of which 55 intact and 17 fragmentary, which is a quite remarkable density of remains. This stratigraphic unit underwent two radiocarbon datings, one of which was performed directly on the grape pips, yielding a chronological range of 1445 to 1192 (cal 2σ) BCE (SI. Table S2). All the whole pips were subjected to GMM analysis and 10 of them were selected for palaeogenetics.

## Results

### Geometric morphometrics (GMM)

**Figure 2:**
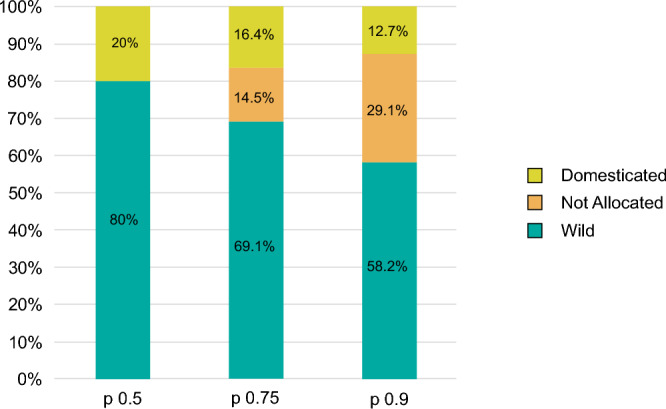
GMM—distribution of archaeological pips according to the allocation to the wild or domesticated compartment.

The linear discriminant analysis (LDA) performed on a balanced collection of modern wild and domestic grape pips allowed to classify a majority of the archaeological pips in the wild morphotype. The domestic morphotype is nevertheless clearly identified. With a probability threshold of p ≥ 0.75, 38 grape pips were assigned to the wild group (69.1%), 9 (16.4%) to the domestic morphotype and 8 (14.5%) remained unclassified (Fig. 2). It must be stressed that, although it is in the minority, the proportion of pips assigned to the domestic type is always significantly greater than the error of the model, regardless of the probability threshold chosen (SI. Table S3). GMM thus provide a robust identification of the domestic grapevine in Middle-Late Bronze Age Pertosa.

Despite their small number, the 9 pips identified as domesticated by this first LDA with p-values ≥ 0.75 were submitted to a second LDA performed at cultivar level (SI. Table  S4). They are allocated to 8 different modern cultivars but the p-value is over 0.75 in only 4 cases. The highlighted cultivars are mostly of eastern origins (Balkans and Caucasus/Near east areas). Only «Bourboulenc» is a traditional French white wine variety. It should be noted that a minor proportion of archaeological pips has previously been assigned to this cultivar morphotype in studies performed in Georgia^29^ and in the Aegean area^44^. Although these results are poorly representative due to the limited number of pips of the domesticated morphotype, we decided to include them in our study anyway as we have the possibility to compare them to aDNA results.

### Ancient DNA

Shotgun sequencing demonstrated that nine of the ten tested seeds had at least 0.2% endogenous DNA (range = 0.00–4.48%; mean = 2.36%) and the expected patterns in cytosine deamination and DNA fragmentation (SI. Fig. S2). Damage-repaired libraries were prepared for the promising samples and enriched for SNPs present in the GrapeReSeq panel, reaching a mean depth of coverage on the SNP loci of 1.1–22.0. An analysis of the relative frequency of alleles at the SNP loci demonstrated these specimens contained < 20% paternal DNA (SI. Fig. S3), consistent with DNA preservation observed in other archaeological grape seeds^30^. Five samples yielded sufficient on-target data to call genotypes at > 1600 SNP loci, following a conservative approach that ignores sites with insufficient data to differentiate between true heterozygous sites and homozygous sites with limited contributions from paternal DNA^31^ (see SI).

**Figure 3:**
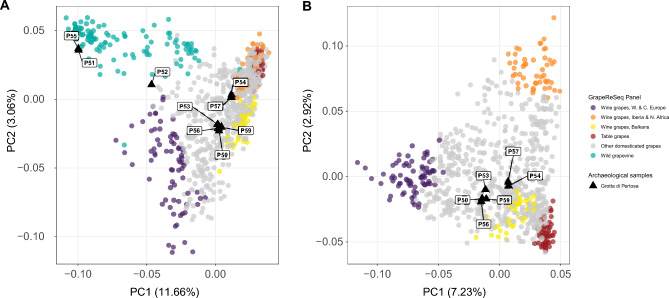
Genetic affinities between pips excavated at Grotta di Pertosa and modern *V. vinifera* accessions. (**A**) PCA with archaeological samples projected on the diversity of wild and cultivated grapevines, with domesticated grapes colored according to the four main ancestry clusters identified by Laucou et al.^6^. (**B**) PCA of putatively domesticated archaeological samples projected on the diversity of domesticated grapevines in the GrapeReSeq panel.

A principal component analysis (PCA) of modern grapevine diversity^45^ projects two archaeological samples among wild *V. vinifera sylvestris* (pips P51 and P55), one near the boundary of wild and domesticated accessions (P52), and six within the PCA-space of domesticated grapevines (Fig. 3A). PCA was repeated using the cultivated panel and the pips affiliated with domesticated grapevines (Fig. 3B), revealing two clusters of the Grotta di Pertosa samples with none directly associated with the four groups defined by Laucou et al*.*^46^, but all near the cluster of modern grapes from the Balkans used for winemaking.

**Figure 4:**
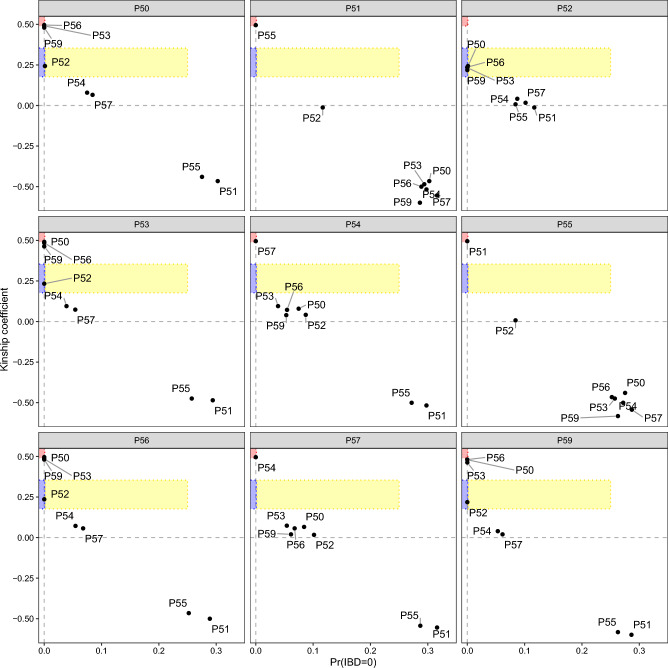
Relatedness among the Grotta di Pertosa samples. Relatedness metrics were calculated using ngsRelate in a genotype likelihood framework based on allele frequencies of the GrapeReSeq diversity panel. For legibility, pairwise comparisons are shown in separate panels for each sample. The software calculates two metrics that distinguish close relationships in grapevine: the proportion of sites where samples share zero alleles identical by state (IBS0) and the KING kinship coefficient. Clonal (monozygotic twins) relationships fall within the red square(K ≥ 0.49 and IBS0 ≤ 0.001), parent–offspring relationships fall within the blue area (0.177 < K < 0.354 and IBS0 ≤ 0.001), siblings/highly related samples fall in the yellow area (0.177 < K < 0.354 and IBS0 ≤ 0.25).

Using the called genotypes, KING found no kinship relationships between the archaeological samples and modern accessions, however it detected kinship within Grotta di Pertosa specimens: one parent–offspring relationship between P50 and P52 and a clonal relationship between P54 and P57. Kinship among all archaeological samples was further explored in a genotype likelihood framework, with ngsRelate identifying the three clusters observed in PCA to be genetic clones: P50 identical to P53, P56 and P59, P51 identical to P55, and P54 identical to P57 (Fig. 4). The software also identified all four members of the first clonal group to be in a parent–offspring relationship with P52.

Genetic distances between modern samples and the four Grotta di Pertosa genotypes were consistent with the PCA plot. The first clonal group (P50, P53, P56 and P59) has the closest genetic affinity with domesticated vines, with the closest being ‘Landroter’, a variety identified as admixed by Laucou et al*.*^46^. The most closely related non-admixed variety is ‘Gouais blanc’, which is a member of the Balkan wine group. The second clonal group (P51 and P55) has close relationships with many *sylvestris* accessions, with the highest in vines from Slovakia and Germany. The third clonal group is mostly closely related to admixed grapes, with the closest being Greek variety ‘Muscat à petits grains blancs’. P52, the only specimen not a part of a clonal group, has genetic affinity to many wild and domesticated grapes, with the closest being ‘Riesling bleu’, but with nearly the same genetic distance to a wild vine from Algeria. While these genetic links show affinity toward the local region, the specimens have even closer genetic links to other archaeological samples. In particular, the first clonal group and P52 show close relationships to Roman seeds from Terrasses de Montfau from southern France and the third clonal group is related to early Roman seeds from Mas de Vignoles XIV near Nîmes^31^ (SI Table S5).

**Figure 5:**
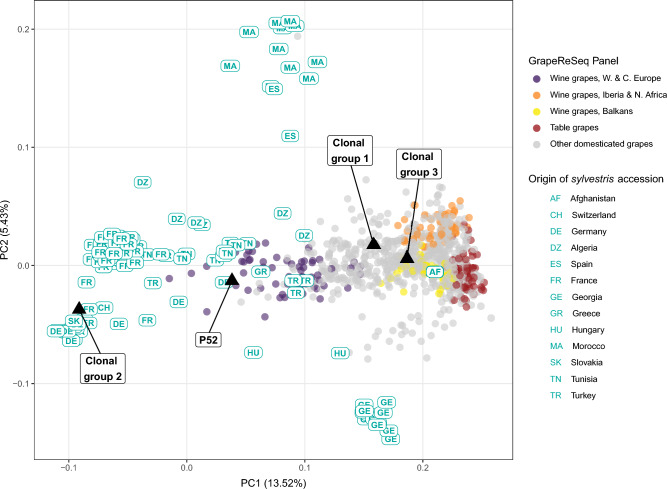
PCA of domesticated and archaeological samples projected onto the diversity of *sylvestris* accessions.

Following the approach by Myles et al*.*^47^, the deeper history of the cultivated vines was explored in PCA by projecting cultivars and archaeological samples onto *sylvestris* diversity (Fig. 5). Consistent with previous observations, most modern cultivars fall in a space separating wild grapevines from Turkey and Afghanistan. Although the reference database has few *sylvestris* accessions from the eastern part of grapevine’s native range—notably lacking samples from Armenia, Azerbaijan, and Pakistan—the analysis recapitulates Myles et al*.*’s^47^ findings, wherein it was argued that the cultivated genepool largely descends from wild grapevines in West Asia. Grotta di Pertosa Clonal groups 1 and 3 are projected into the same space as modern cultivars, indicating they have little or no gene flow from *sylvestris* populations in Greece, Italy, or other parts of Western Europe. In contrast, Clonal Group 2 falls among *sylvestris* accessions from Slovakia, Germany, and France, potentially consistent with a local Italian origin. The admixed P52 specimen falls midway between its presumptive domesticated parent and the wild grapevines of Western Europe. P52’s position is shared by some cultivated vines used for winemaking in Western and Central Europe, and these varieties have been shown to be the product of crossing with local *sylvestris*, however none appear to result from an independent domestication.

## Discussion

### Early viticulture in Bronze Age Italy

The harvesting of what were in all probability wild grapes is sporadically attested in Southern Italy since the Upper Palaeolithic^48^ and the Mesolithic^49^ within two sites. However, it is during the Neolithic that seeds of *Vitis vinifera*, plausibly still wild, become more common in the archaeobotanical assemblages of Italian sites^34,50–54^. This is more clear in the northern regions, but is also recorded in the south, where 5 sites are known. Domesticated grapevine was identified by morphometric and paleogenetic analyses of desiccated, uncharred pips from a Late Neolithic layer in Grotta della Serratura^55^, in the Campania region. However, no direct radiometric dating was performed and no explanation is provided to explain the preservation of uncharred pips in a dry site. This result should therefore be considered with caution until it is supported by direct dating and/or by matching results from other sites.

During the Early Bronze Age several pile-dwelling settlements and other wetland sites in northern Italy have yielded rich assemblages of grape pips, all regarded as wild^56–59^. It is in the Middle Bronze Age that a clear increase in attestations is seen throughout the peninsular territory^60–62^, and considered as possibly linked to wine production^60,63,64^. Basing mainly on biometric indices^18–20^, but also on simple morphological observations, some authors suggest the appearance at this time of the first domesticated types^60,65^ which would be more frequently attested during the Late and Final Bronze Age^60,66–74^.

This alleged general trend is primarily based on the evidence from the northern and central Tyrrhenian regions of the peninsula, while in the southern and Adriatic regions the evidence is much less abundant^34^ and less extensively studied. Nevertheless, in this phase, grape pips have been discovered in 21 southern sites (Fig. 1, SI Table S1). Unfortunately, the high number of sites does not correspond to a large quantity of pips discovered, and those that have been recovered are often fragmentary.

The discrepancy between the quantity of the evidence of vines between the northern/central Tyrrhenian area and the southern/central Adriatic area of the Italian peninsula is partly due to the fact that the former hosts numerous sites located in wetland environments (i.e. pile-dwelling settlements near the Alps and on the volcanic lakes of central Italy, as well as the Terramare culture in the Po plain), which, given the characteristics of their sediments, conserve organic remains even in the absence of combustion. In contrast, in the south and the islands, mainly charred grape pips are discovered, their preservation probably being the result of fortuitous and unusual conditions. However, an increase in grapevine interest has also been recorded by pollen analyses conducted on cores taken from sites in both Apulia, in the Alimini Piccolo lake^75^ and the Salpi lagoon near Coppa Nevigata site^76^, and in Campania, in the Gulf of Gaeta^77^. In addition, three discoveries dated to the Middle Bronze Age could constitute indirect evidence of viticulture. These are the 21 vine branches recovered from a well in the Strepparo e Cento Moggie district in Capua^78^, together with various anthracological remains of *Vitis vinifera* discovered in workshops in Coppa Nevigata^79^ and Tufariello^80^. The cited cases have been interpreted as evidence of pruning, indicating the existence of practices associated with the care and management of grape vines from as early as the Middle Bronze Age.

### Diffusion of grape varieties and allochthonous input

Considering the historical period we can observe a chronological coincidence between what we believe to be the first evidences of viticulture in Southern Italy with the arrival of Mycenaean people on its coasts. Given that a gene flow between the vines of Greece and southern Italy took place at some point in history^81,82^, it may be wondered whether viticulture in Italy was in some way triggered or accelerated by allochthonous inputs from the eastern Mediterranean, at its very dawn. The answer is not obvious. In order to pursue this aim it is worthwhile to establish certain key points regarding Mycenaean visits to the Italian coasts during the Bronze Age (SI Fig. S1), which is a fairly complex theme^83^. The relationships between the Mycenaeans and the local populations differed from region to region and changed over time, ranging from occasional and sporadic visits to consolidated trade relations and more complex forms of cultural integration, with the imitation of products and models^84–87^. The traffic between Southern Italy and the Aegean area may have included food products that leave highly labile traces in the archaeological record which is significant and opens up new spaces for reflection. This may have included both livestock^88^ and agricultural products. Remains of *Triticum timopheevii* found in Grotta di Pertosa could constitute a clue in this sense^89^. Indeed this is the only case for this species to be identified in southern Italy, whereas, as far as we know, its distribution range goes from Anatolia to the Balkan peninsula up to the Alpine area and Central and Eastern Europe^90–95^.

Chronologically, the Mycenaean contacts start to become evident in about 1700 BCE. Significant evidence of strict contacts has been found during the subsequent phase, i.e. LH III A, corresponding to the Apennine facies in southern Italy (about 1450–1300 BCE). After this period the Mycenaean palatial system collapsed, although relations between the eastern Mediterranean and Southern Italy continued, as attested by the abundant discoveries of LH III B-C materials in Sub-Apennine contexts (Late Bronze Age). The period saw the rise of new cultural manifestations such as the imitative ceramics known as Italo-Mycenaean products^83–85,96,97^.

Grotta di Pertosa is located in an area not directly exposed to Mycenaean traffic, but several archaeological indicators (i.e. faience beads, bronze artifacts and weapons, imported lithic raw materials, *timopheevii* wheat) show a certain degree of integration in a network of contacts on the regional and transregional scale^41,89^. Furthermore sporadic Aegean ceramic fragments have been discovered in underground funerary contexts very close to Grotta di Pertosa^98,99^.

**Figure 6:**
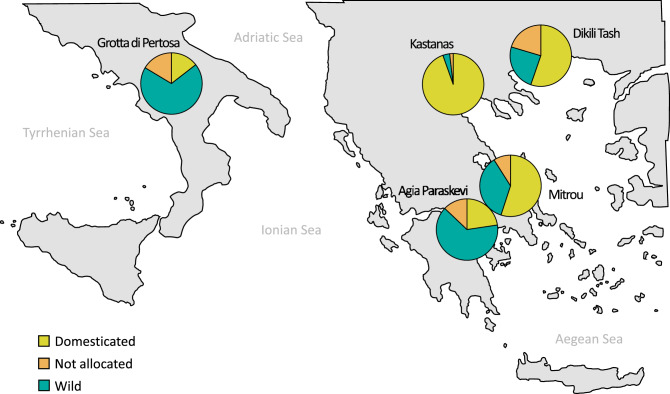
Distribution of archaeological pips according to the allocation to the wild or domesticated compartment (p 0.75) and to their geographic location (Greek sites data after Pagnoux et al. 2021^44^).

Thanks to new data on the remains of grape pips from Greek contexts described in a recent publication^44^, it is possible to compare our results with those more or less coeval sites, analysed with the same geometric morphometric method (Fig. 6). Of the four Bronze Age sites considered in that study, the one with a wild/domestic morphotypes ratio comparable to Pertosa is Agia Paraskevi, which is few centuries older. The other three sites, contemporary to Pertosa (i.e. Dikili Tash, Mitrou and Kastanas) show an already well established viticulture with domestic morphotypes representing the 55 to 95% of the total grape pips analyzed. Unfortunately the pips from Greek sites are charred so it is not possible to verify the existence of any genetic correlation with our sample. A GMM comparison of the domestic grape pips of Kastanas and Mitrou with modern varieties shows that some of them are very similar to modern cultivars grown in Turkey, the Levant and the Caucasus. The authors did not exclude the introduction of domestic varieties from the East and their potential hybridisation with local varieties^44^.

Similar considerations can be made on the basis of our results from GMM and aDNA on the Pertosa samples, in which a relationship seems to emerge between the introduction of already domesticated varieties (likely from Balkans and Greece) and their hybridisation with local wild grapes. This previously unknown data for the western Mediterranean, supported both by GMM and ancient DNA results, seems to be correlated to the first contacts with Mycenaean people, who have probably already experimented with this model and spread it where there is suitable local wild material.

### Use of local wild grapes

The results of the aDNA analyses showed no evidence of independent domestication of local wild grapes. Nonetheless, a parent–offspring relationship emerged between a domestic/wild hybrid individual (P52) and a domesticated clonal group (P50, P53, P56 and P59). Besides GMM showed that vines producing morphologically wild pips were surely exploited. This highlight the contribution of wild grapes in the early viticulture of this area. We cannot state whether the crossing was voluntary or accidental and we do not know the extent of the phenomenon. However, the presence of clonal groups and hybrids within the sample, as well as the coexistence of wild and domesticated grapes, leads us to suppose that this first stage of viticulture in Middle to the Late Bronze Age Southern Italy included both asexual and sexual propagation of the vines, as well as the introduction of non-native varieties and the use of local wild vines. Selective pressure had to be rather mild and this could explain the proportions between domesticated and wild morphotypes in the Grotta di Pertosa assemblage. Indeed, GMM analyses shows that the percentage of domestic morphotypes reaches 16.5%, while the wild ones are the 69.1%.

Such a consideration is in line with what has emerged from recent studies^27,44,100^, according to which, viticulture in its initial stage, requiring maintenance of the plants involving low selective pressure and occasional recourse to sexual reproduction, does not necessarily result in drastic changes in the shape of the seeds. This is seen only gradually, in a non-linear manner, as the selective pressure exerted on the plants by human beings increases. It is manifested on the archaeobotanical level by mixed assemblages composed of variable percentages of fully domestic types, wild types and weakly selected types, as in the case presented here, according to a trend that persists even in subsequent historical periods in other areas^21^. Our study adds a piece to this reconstruction and suggests that, in some cases, the less clear morphotypes could represent wild-domestic hybrids, rather than weakly selected types.

## Conclusion

This work has allowed us to establish some fixed points on the diffusion of viticulture in the western Mediterranean. The presence of domestic vines in the south of the Italian peninsula between the Middle and Late Bronze Age, already presumed from other archaeobotanical indicators, has been confirmed beyond any doubt. In this historical phase the indigenous Italic communities experience a growing economic and social complexity and contacts with Mycenaean peoples constitute an important factor of cultural contamination. Within this framework, the introduction of domestic grape varieties from the Aegean area, as well as the growing interest in the cultivation of vines, could represent a reflection of the adhesion by local élites to a system of uses and values spread by Mycenaean sailors throughout the Mediterranean. Local societies, however, do not passively absorb the new models, but make them their own and reinterpret them. It is suggestive to note how this mechanism, observed for some aspects of material culture, is in a certain way also valid for viticulture practices: the domestic grapes introduced from the East, in fact, hybridize with the local wild ones, harvested and exploited in this region for thousands of years, originating something new.

## Materials and methods

A total of 55 grape pips from Grotta di Pertosa, dated to the latter period of the Middle Bronze Age to the beginning of the Late Bronze Age (from 1440 to 1200 BCE), were analysed in this study by geometric morphometrics. Although the total number of specimens discovered in the site was greater, only the pips that were perfectly preserved could be taken into account. All the grape pips analysed were waterlogged and were from an inhabited context, precisely from a single stratigraphic unit, formed by the collapsed floor of a pile-dwelling. From these, a group of ten seeds, showing a certain degree of shape diversity, were selected for paleogenetic analysis. All plant experiments were carried out with relevant institutional, national, and international guidelines and legislation.

### Geometric morphometric analyses

Geometric morphometrics were based on seed outline analysis using the Elliptic Fourier Transform (EFT) method, which is by now widely known in the literature and has been consolidated over the last decade^23,28,44,95^. The 55 selected grape pips were photographed in dorsal and lateral views using a stereomicroscope (Olympus SZ-ET) connected to a digital camera (Olympus DP12). Subsequently, these images were semi-automatically converted into black shapes on white backgrounds by means of photo editing software. All the morphometric analyses were then performed using the Momocs package^101^ in R environment (R Development Core Team 2021). The x and y coordinates of 360 equidistant points were sampled along the outline of each profile. The outlines were normalized by centring and scaling according to their centroid size and the first point was positioned above the centroid. The computation of EFT allows to decompose the seed outlines into a harmonic serie of trigonometric functions described by numerical coefficients that can be used as shape descriptors in statistical analyses. Only the 6 first harmonics were considered, as a good compromise between the need to precisely describe the shape and to minimise the errors^24^. Each harmonic is associated with 4 coefficients, and thus each outline is described by 24 coefficients, bringing the total number of coefficients used to describe each individual seed to 48.

### GMM statistical analyses

All statistical analyses were performed with the MASS package^102^. Based on the 48 shape descriptors, the morphology of the 55 archaeological seeds was characterized by comparison to modern populations of grape pips using Linear Discriminant Analyses (LDA). A two-step approach was applied using two nested LDAs and the modern reference collection of wild grapevines and cultivated varieties of ISEM^29,44^. These wild grapes and modern cultivars are originating from various countries from Western Asia to Central and Western Europe and around the Mediterranean. A first LDA was performed to identify the wild or domesticated status of the archaeological pips. The archaeological pips were compared to a balanced modern collection of randomly selected pips of wild (N = 2430) and domesticated (N = 2430) grapes. The power of this method in discriminating between domestic and wild morphotypes has been demonstrated in previous studies^24,29^. Using this balanced reference collection and leave-one-out cross-validation 95.7% of the pips are correctly classified according to their wild or domesticated status^29,44^.

The archaeological pips identified as domesticated (p value ≥ 0.75) by this first LDA were then submitted to a second LDA designed to compare their morphology to modern varieties. This second LDA was based on a balanced collection composed of 280 traditional cultivars whose main agronomic traits and areas of origins are known, with ca 30 pips per cultivar. In this case, leave-one-out cross-validation allows the correct cultivar identification of 77.18% of the pips.

### aDNA methods

Ancient DNA was recovered and analyzed following methods developed by Ramos-Madrigal*, *et al*.*^31^, with details described in supplementary information and data. Ultrashort DNA was extracted from ten individual seeds^103^, converted to Illumina libraries using a single-stranded DNA preparation^104^, treated with uracil–DNA–glycosylase to repair DNA damage^105^, and enriched using an in-solution kit which targets 10,000 single nucleotide polymorphisms (SNPs)^31^. Sequencing data were processed in the PALEOMIX pipeline following recommendations for degraded DNA^106^. Genetic structure and relatedness were investigated in the context of a grapevine reference panel of 783 cultivars^46^, 112 wild accessions^107^, and 31 archaeological samples^29,31^, using smartpca^45^, KING^108^, and ngsRelate^109^.

### Supplementary Information

Supplementary Information 1.

Supplementary Information 2.

Supplementary Information 3.

Supplementary Table S5.

## Data Availability

The datasets generated and/or analysed during the current study are available in the NIH repository, https://dataview.ncbi.nlm.nih.gov/object/PRJNA999173?reviewer=j47gnuhdvgai0tj72ljisb78vq.
