# Supplementary material for: Disentangling the origins of viticulture in the western Mediterranean
Source: Sci Rep. 2023 Oct 12;13:17284. doi: 10.1038/s41598-023-44445-4 (PMC10570292; doi:10.1038/s41598-023-44445-4)
Supplement: Supplementary file 3 — Supplementary Information 3. [file 41598_2023_44445_MOESM3_ESM.pdf]

Supplementary Information for
Disentangling the origins of viticulture in the Central Mediterranean.

Francesco Breglia[1,4*], Laurent Bouby[2], Nathan Wales[3], Sarah Ivorra[2], Girolamo Fiorentino[4].

[1] Department of Geosciences, University of Padua, Padua, Italy - 35131

[2] Institut des Sciences de l'Evolution, University of Montpellier, Montpellier, France - 34095

[3] Department of Archaeology, University of York, York, UK - YO1 7EP

[4] Cultural Heritage Department, Laboratory of Archaeobotany and Palaeoecology, University of Salento, Lecce, Italy - 73100

*Francesco Breglia

Email: francesco.breglia@unipd.it

This PDF file includes:

Supporting text
Figures S1 to S3
Tables S1 to S4
SI References

Supporting Information Text

Supplemental Methods

DNA extraction. Ancient DNA from ten waterlogged grape pips from Grotta di Pertosa was recovered and analyzed following grapevine palaeogenomic methods developed by Ramos-Madrigal, et al. [1] (Fig. S2). Archaeological seeds were processed in a dedicated palaeogenomics facility at the University of York. The facility adheres to recommendations for ancient DNA research [2], including physical separation from post-PCR laboratories, positive air pressure, UV-C irradiation of benches and laminar flow cabinets, use of full-body suits by laboratory personnel, and frequent sterilization of surfaces.

Archaeological seeds were processed individually to recover a single genetic profile. Samples were bleached in 5% NaClO solution for 30 seconds to reduce modern DNA contamination and then rinsed in molecular biology grade water. Seeds were manually crushed with sterile micropestles and further homogenized in PowerBead tubes (Qiagen catalog number 13117-50) by oscillating in a Retsch Mixer Mill MM200 for 10 minutes at 25 Hz.

DNA was isolated from the specimens and two extraction controls using a protocol designed for archaeobotanical remains [3]. The procedure consists of an overnight incubation in a lysis buffer, a phenol-chloroform DNA extraction, and a subsequent purification in a MinElute column (Qiagen catalog number 28004) with an optimized GuHCl binding buffer to retain ultrashort DNA molecules [4]. The DNA eluates were slightly pigmented, potentially caused by the co-extraction of polyphenols from the pips or humic acids from the archaeological sediment. As these compounds may inhibit enzymatic reactions during library preparation or PCR amplification [5, 6], the pigmented DNA extracts were further purified with OneStep PCR Inhibitor Removal columns (Zymo Research catalog number D6030). The manufacturer's recommendations were followed, except that the 30 µl DNA eluate added to the OneStep PCR Inhibitor Removal columns was below the suggested 50–200 µl input volume. DNA concentrations were measured with a Qubit dsDNA HS assay kit (Invitrogen catalog number Q32854).

Illumina library preparation. DNA was converted to Illumina libraries using the Santa Cruz Reaction (SCR) single-stranded DNA protocol for ancient DNA [7]. SCR libraries were prepared in two rounds: screening libraries to estimate endogenous content and observe damage profiles and higher-complexity, damage-repaired libraries for enrichment of SNP loci.

The screening libraries were prepared on 5 µl of purified DNA (~17% of total extract volume). One SCR library control was also prepared on 5 µl of molecular grade water to monitor contamination. The amount of SCR adapter-splints and extreme thermostable single-stranded DNA binding proteins (ET SSB) were varied according to the amount of input DNA, following the suggested tiers in the SCR protocol. A real-time quantitative PCR assay was used to estimate the number of cycles needed for indexing PCR [8]. The screening libraries were PCR-amplified in 25-µl reactions, containing 8–12 µl of library, 1×AmpliTaq Gold buffer, 2.5 mM $MgCl_2$, 200 µM dNTPs, 1 U AmpliTaq Gold 360 polymerase, 1 µM forward primer with 8-mer sample-specific index, 1 µM reverse primer with 8-mer sample-specific index, and 0.4 mg/mL bovine serum albumin. The PCR cycling conditions were 10 minutes at 95°C to denature DNA and activate the polymerase, 8–18 cycles of 95°C for 30 seconds, 60°C annealing for 30 seconds, and 72°C extension for 60 seconds, and a final extension of 72°C extension for 7 minutes. The amplified libraries were purified with homemade SPRI beads [9] and quantified with a TapeStation High Sensitivity D1000 assay (Agilent catalog number 5067-5584). The indexed libraries, including both extraction controls and the library control, were pooled and sequenced on a partial lane of an Illumina HiSeq 4000 in paired-end mode for 150 cycles at the Novogene facility in Sacramento, California.

The screening data demonstrated nine archaeological specimens contained more than 0.1% endogenous DNA and were progressed to the second phase of library preparation. The remaining raw DNA (25 µl) was treated with USER enzyme (New England BioLabs) to remove uracil residues, following the approach of a full uracil–DNA–glycosylase (UDG) treatment [10], with adaptations for

the SCR protocol. The USER enzyme treatment was performed in 50-μl reactions containing 1× Cut Smart buffer and 3U USER enzyme, using an incubation of 37°C for three hours. The USER-treated DNA was purified in MinElute columns with the optimized GuHCl binding buffer to retain ultrashort DNA molecules [4]. The DNA was eluted in 20 μl to match the maximum input volume of the SCR library protocol. The entire volume of USER-treated DNA was converted to SCR libraries as described previously. The indexing PCR steps were performed as above, except that the PCR reaction size was 100 μl, the amount of input library was 48 μl (the entire volume), and 8–10 PCR cycles were used.

DNA Enrichment of SNP loci. The high-complexity, damage-repaired SCR libraries were enriched using a custom myBaits target capture kit produced by Daicel Arbor Biosciences. The enrichment kit contains RNA probes to target 10,000 SNPs [1], allowing for the identification of cultivated varieties and analysis of population structure in wild and domesticated grapevine accessions [11].

The nine SCR libraries were enriched using three multiplex reactions, with each reaction containing three indexed libraries. The libraries were combined into three pools based upon similar endogenous content, with input volumes varied to reach similar amounts of input DNA.

Manufacturer's recommendations were followed for the enrichment and PCR steps, using myBaits manual (version 5.01) and the optional "High Sensitivity Protocol" for ancient DNA. For the blockers mix setup, the suggested set of blockers for plants were used: 5.0 μl of Block O and 0.5 μl of Block X. Two rounds of enrichment were used, with a hybridization temperature of 55°C during both overnight incubation periods. After the first enrichment, libraries were amplified 14 cycles, as per the manual, but following the second enrichment, libraries were amplified 10–14 cycles to reach the necessary amounts for sequencing (Fig. S2). The enriched libraries were quantified and sequenced on a portion of a partial lane of an Illumina HiSeq 4000 in paired-end mode for 150 cycles at the Novogene facility in Sacramento, California.

Reference datasets. Genotypes of modern grapevines in the GrapeReSeq genetic diversity panel provide the main context to interpret the Grotta di Pertosa archaeological samples. The GrapeReSeq panel consists of 783 domesticated cultivars and 112 wild grapevine accessions [11, 12], each genotyped at >10,000 SNPs using Illumina chip technology. The genotype data were taken from the respective repositories and examined to confirm SNP coordinates in the grape nuclear reference genome 12×.v2 [13]. Specifically, the metadata for the cultivar dataset (available at https://urgi.versailles.inra.fr/Species/Vitis/Data-Sequences/Genotyping-data) was used to localize Illumina chip SNPs by taking the flanking sequences and aligning them to the reference genome with BLAST+ (version 2.10.0) [14]. SNPs were retained when they could be unambiguously aligned to the reference genome and matched the genetic positions listed in the metadata. Likewise, the PLINK files provided in the wild accession database were examined to confirm that the positions and alleles matched those in the cultivar database. After excluding SNPs which could not be unambiguously localized, the cultivar dataset contained 8773 SNPs and the combined cultivar and wild dataset contained 8191 SNPs.

In addition to the modern grapevine accessions, the Grotta di Pertosa samples were compared to two published archaeological datasets which utilized enrichment with the custom myBaits in-solution kit. The first archaeological dataset consists of twenty-eight grape seeds excavated in France dating to the Iron Age to medieval period [1] and the second consists of three seeds excavated from Georgia and dating to the past 600 years [15].

Ancient DNA data analysis. The Grotta di Pertosa sequencing data were processed in the PALEOMIX (version 1.2.13.4) bioinformatic pipeline [16], following established practices for ancient DNA data. AdapterRemoval v2.3.0 trimmed Illumina adapters and collapsed overlapping paired reads, retaining trimmed reads which were at least 25 bp. Trimmed reads were mapped to the grapevine 12×.v2 nuclear and plastid genomes [13, 17], using BWA aln (version 0.7.17) with the seeding disabled [18]. PCR duplicates were removed with MarkDuplicates (http://picard.sourceforge.net) and reads with mapping qualities below 30 were removed. Reads

were realigned around insertions and deletions using GATK (version 3.8) [19]. Damage profiles were plotted using mapDamage (version 2.0.9) [20].

The libraries which had been enriched for SNP loci were used in population and relatedness analyses. The UDG treatment removed damaged nucleotides from the center of the DNA molecules, but damage was still present (>0.01 frequency) in the first and last three nucleotides. To reduce the impact of damage in analyses, the trimBam function of BamUtils (version 1.0.14) [21] was used to trim three nucleotides from the 5' and 3' end of each read.

The raw data from the published datasets from French [1] and Georgian [15] archaeological samples were processed following the same methods as those used on the Grotta di Pertosa data, except for the steps to correct DNA damage. As previous projects did not implement UDG treatment, the damage patterns were corrected using two steps. First, the base qualities of likely damaged positions were rescaled using mapDamage. Subsequently, five nucleotides were trimmed from the 5' and 3' end of each read using BamUtils. The depth of coverage on the GrapeReSeq SNP loci was calculated with SAMtools (version 1.10) [22] after filtering PCR duplicates, removing poorly mapped reads, and correcting for damage.

Ramos-Madrigal, et al. [1] demonstrate that archaeological seeds contain primarily maternal DNA, i.e., the genotype of the vine on which the berry was growing. Paternal DNA is present in fresh seeds in the endosperm and embryo, but this paternal contribution has been observed to be <20% in other archaeological seeds and can be excluded to infer the maternal genotype at many loci [1]. The amount of paternal DNA was investigated here by examining the relative frequency of the reference allele at SNP loci where both alleles were observed. GATK's HaplotypeCaller (version 4.1.3) was used to calculate the number of reads corresponding to the reference and alternative alleles. The paternal DNA analysis was restricted to loci with a minimum depth of coverage of ten reads in R (version 4.2.0) [23].

Population structure was examined using a principal components analysis implemented in the smartpca function of the EIGENSOFT (version 7.2.1) package [24, 25]. This approach uses psudeo-haploid genomes to accommodate samples with highly variable sequencing depths and missing data. Mapped reads for the enriched libraries were randomly sampled at the GrapeReSeq SNP loci using SAMtools and pileupCaller (https://github.com/stschiff/sequenceTools), using a minimum base quality of 30, minimum mapping quality of 30, and disabling the base alignment quality computation. The files were merged with pseudo-haploid versions of the reference panel using EIGENSOFT. The archaeological samples were projected onto the modern reference panel in smartpca using the autoshrink mode. PCA data were processed in R (version 4.2.0) [23] using the ggplot2 package [26].

Genotype calling was performed on well-covered sites that should be minimally impacted by limited paternal DNA contributions. A HaplotypeCaller GVCF file for each sample was processed using BCFtools (version 1.15) [22], setting genotypes to missing data if they had a depth of coverage less than ten reads or unusually low or high frequencies of the alternative allele ($0<AF<0.2$ or $0.8<AF<1$). That is, well-covered loci with exclusively one allele were genotyped as homozygous and putative heterozygous sites were genotyped as heterozygous if the frequency of the alternative allele was 0.2–0.8. While this approach discards data some potential true heterozygous sites, the retained data are of a higher quality. It is also worth noting that a small proportion of sites which are called as homozygous are likely heterozygous, due to the random sampling of alleles at a relatively low depth of coverage. For SNP sites with 10× coverage, the binomial distribution estimates that 0.195% of true heterozygous sites would be genotyped as homozygous. Still, the absolute number of these genotyping errors are expected to be low; for example, for modern samples in the GrapeReSeq dataset (mean heterozygosity rate of 0.32), this would correspond to approximately six heterozygous loci genotyped as homozygous in the 10,000 SNP panel. Furthermore, the kinship software used in this project was selected to accommodate low levels of error (e.g., enrichment biases, sequencing errors, genotyping errors on the Illumina SNP chip).

Kinship between samples and the GrapeReSeq panel was investigated using two methods: one approach with called genotypes and a second method using genotype likelihoods to accommodate samples with lower coverage. In the first approach, genotypes of individual archaeological samples were merged with the GrapeReSeq panel using PLINK (version 1.9) [27] and processed in KING (version 2.2.8) [28]. In the second approach, genotype likelihoods for the SNP loci were calculated in ANGSD (version 0.930) [29], using the SAMtools genotype likelihood model, a minimum mapping quality of 30, and a minimum base quality of 20. The genotype likelihoods were analyzed in ngsRelate (version 2) [30], assuming outbred individuals and using the allele frequencies of the GrapeReSeq panel. For both the genotype calls and genotype likelihoods, relationships between pairs of samples were identified using the KING kinship coefficient and the proportion of loci where no alleles are shared (zero alleles identical by state: IBS0). The classification of relationships followed previous suggestions to distinguish identical clones ($K \geq 0.49$ and $IBS0 \leq 0.001$), parent-offspring relationships ($0.177 < K < 0.354$ and $IBS0 \leq 0.001$), highly related individuals ($0.177 < K < 0.354$ and $IBS0 \leq 0.25$), and unrelated individuals [1, 11]. The “highly related” category is effectively analogous to sibling relationships, but simulations by Ramos-Madrigal*, et al.* [1] demonstrated that pollination by a closely related vine could impact interpretations if archaeological seeds contain high amounts of paternal DNA contributions.

To further examine genetic distances between archaeological samples and modern accessions, PLINK was used to calculate identity by state (IBS). As several samples were identified as clonal, the data for each clone was merged to increase the number of SNP loci which could be genotyped. Genotype calling was performed on the merged BAM files as previously described. Archaeological samples were included in the analysis if they were genotyped for at least 2000 loci.

Supplementary figures and tables

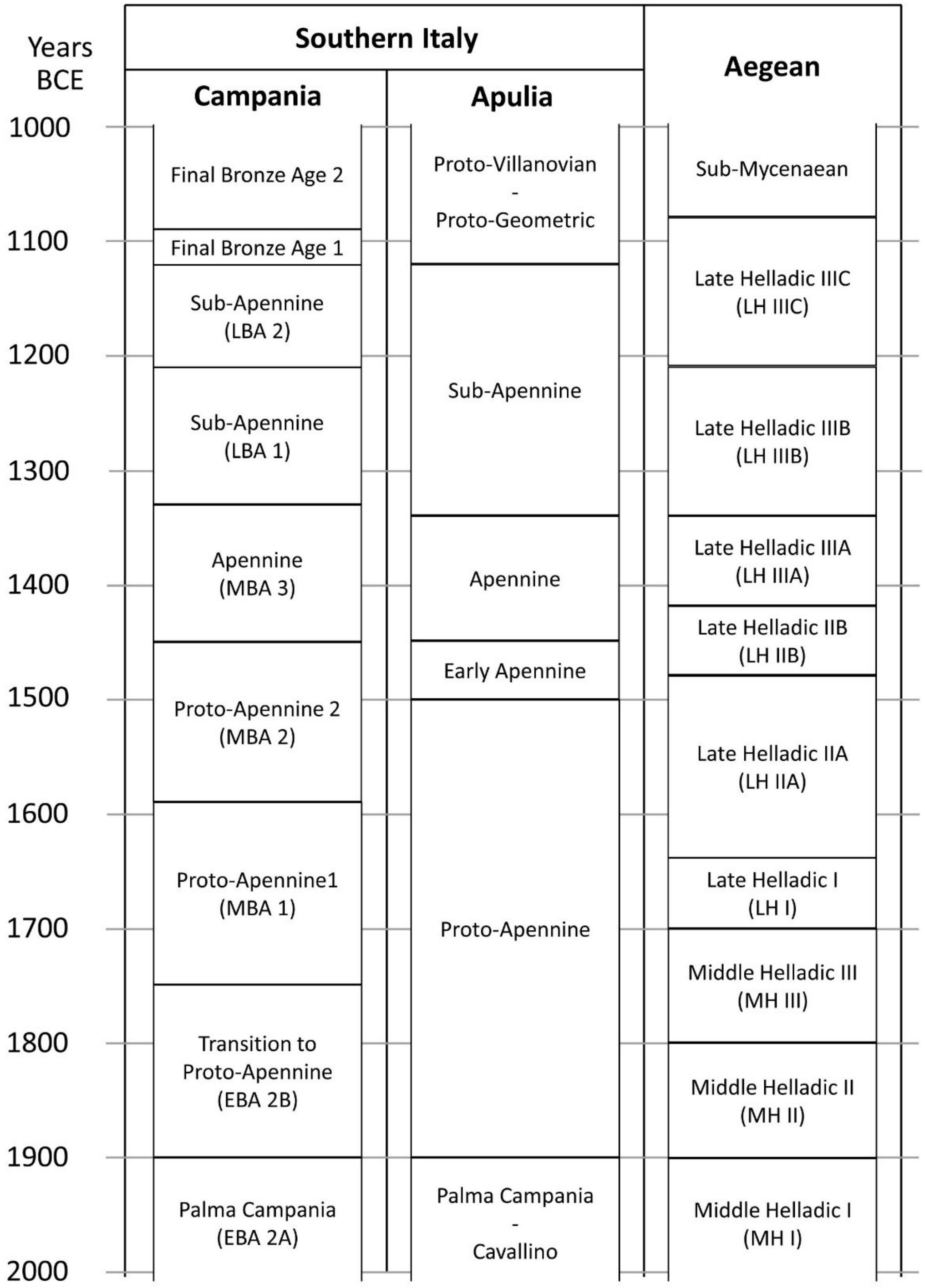

Figure S1. Synoptic chronological table of the Bronze Age archaeological facies in Southern Italy and Greece.

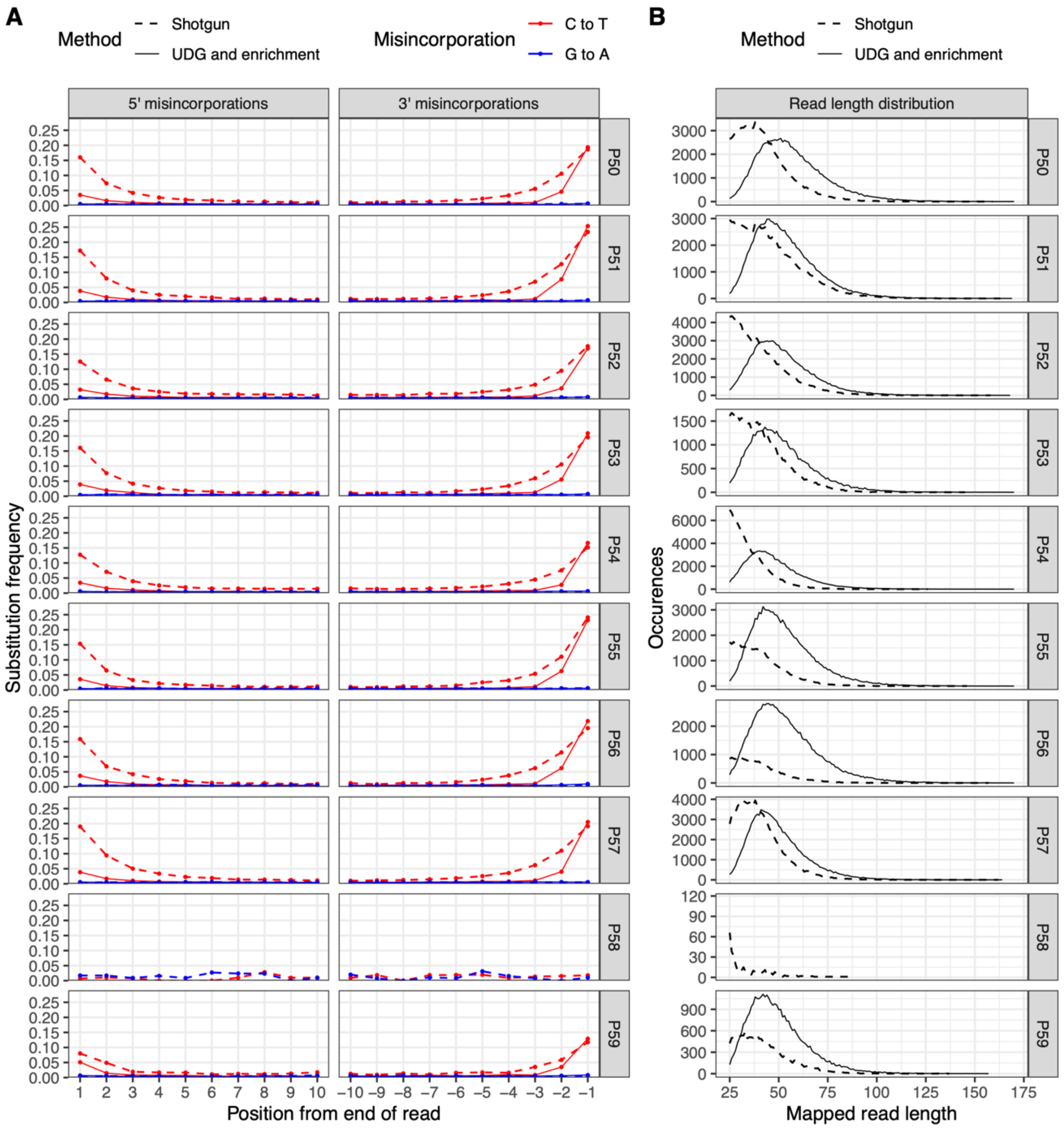

Figure S2. DNA degradation patterns. A. Nucleotide misincorporations at the 5' and 3' ends of mapped reads were identified using mapDamage (20). Shotgun sequencing yielded characteristic patterns of apparent C-to-T transitions at both ends of the reads, consistent with libraries prepared on single-stranded DNA with deaminated cytosine residues (31). The libraries used in the SNP enrichment experiments were prepared on DNA treated with uracil–DNA–glycosylase (UDG) to repair damage. The enriched libraires demonstrated a reduction in damage patterns, particularly in the center of the reads, and remaining damage patterns in the terminal three nucleotides were corrected bioinformatically. As expected for single-stranded DNA libraries, few G-to-A misincorporations are observed. B. DNA length distributions for both sequencing experiments. The shotgun sequencing yielded shorter reads than the enrichment experiment (mean of 40.3 bp and 51.8 bp, respectively). Specimen P58 was not enriched for SNPs as it yielded negligible amounts of endogenous DNA and a damage profile which was not consistent with degraded DNA.

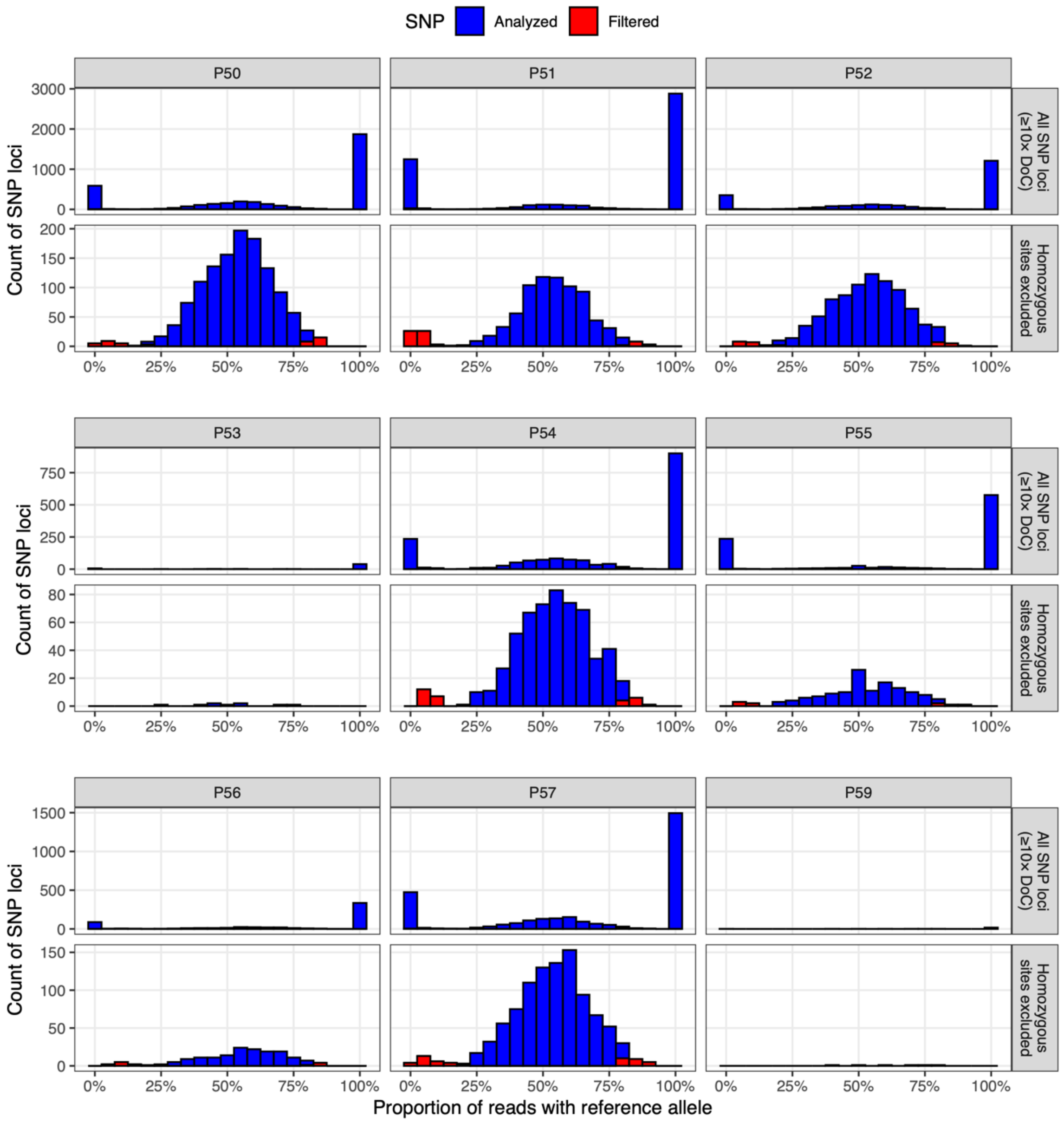

Figure S3. Evidence of paternal DNA. Histograms show the proportions of reads with the reference allele at SNP loci sequenced to a depth of at least 10×. The top panels show all SNPs, including putative homozygous sites, while the bottom panels are restricted to sites where the reference and alternative alleles were observed. Most seeds have evidence of limited contributions of paternal DNA, as seen in the small peak of heterozygous loci where 5–15% of reads contain the alternative allele. This scenario can be interpreted as loci where the maternal genotype is homozygous for the alternative variant and the paternal DNA contains at least one copy of the reference allele. Paternal DNA is likely also responsible for shifting modes above 50%; these loci are consistent with a heterozygous maternal genotype and ~10% paternal DNA with a homozygous reference genotype. Given the evidence for paternal DNA contributions, genotyping was conducted in a conservative manner, following Ramos-Madrigal*, et al.* (1). Specifically, potential heterozygous loci were recorded as missing data if they contained <20% or >80% of one allele (red). Homozygous loci and well-supported heterozygous loci were genotyped and included in downstream relatedness analyses (blue). All read depths were calculated after removing PCR duplicates and reads with low mapping qualities.

Table S1. Bronze Age Vitis vinifera seeds found in southern Italy.

Site	Datation	Total number of remains	Preservation state	Wild type pips	Domesticated type pips	Undetermined	Biometric or morphometric method applied
La Muculufa [32, 33]	EBA	n/a	charred	-	-	-	none
Nola - Piazza d'Armi [34]	EBA	1	charred	0	0	1	none
Nola - Croce del Papa [35]	EBA	3	charred	0	0	3	none
Pompei [36, 37]	EBA	n/a (few)	charred	-	-	-	none
Afragola [38, 39]	EBA	n/a	charred	-	-	-	none
Filo Braccio [40, 41, 42, 43]	EBA/MBA	132 (77 fragm)	charred	43	1	11	Mangafa & Kotsakis [44]
Portella [45, 46]	MBA	9 (6 fragm)	charred	0	1	6	Stummer [47]
				2	1	2	Perret [48]
				3	0	0	Mangafa & Kotsakis
Grotta della Madonna [49, 50]	MBA	1	charred	0	0	1	none
Oratino - La Rocca [51, 52]	MBA (?)	58	dehydrated	0	0	58	none
Vivara - Punta d'Alaca [53]	MBA	9 (6 fragm)	8 charred<br>1 mineralised	0	0	3	Stummer
Tufariello [54]	MBA	1	charred	0	0	1	none
Torre Mordillo [55]	MBA/LBA	1	charred	0	0	1	none
Grotta di Pertosa [56]	MBA/LBA	72 (17 fragm)	waterlogged	38	9	8	GMM ($p \geq 0.75$)
Fonte Maggio [57]	MBA/LBA	3	charred	3	0	0	none
Punta Zambrone [58]	LBA	44 (35 fragm)	charred	0	0	44	none
Roca [59]	LBA	1	mineralised	0	0	1	none
Torre Castelluccia [60]	LBA	4	charred	0	0	4	none
Lipari - Acropoli [43]	LBA	4	charred	0	0	4	none
Broglio di Trebisacce [61, 62]	LBA	5 (1 fragm)	charred	0	0	1 fr	none
			dehydrated	0	0	4	none
Fattoria Mammarella [57]	LBA	1	charred	1	0	0	none
Mokarta [63]	FBA	1	charred	0	0	1	none

Table S2. Radiocarbon datings performed on seeds from the same stratigraphic unit.

Site	Sample ID	Type of sample	Not calibrated dating - BP	δ13C (‰)	Calibrated dating (cal 2σ) BCE
Grotta di Pertosa	LTL19889A	*Triticum dicoccum* (caryopsis)	3092 ± 45	-24.2 ± 0.5	68,2% prob<br>1414 – 1368 BCE (27,9%)<br>1361 – 1295 BCE (40,3%)<br>95,4% prob<br>1445 – 1227 BCE (95,4%)
Grotta di Pertosa	LTL19885A	*Vitis vinifera* (pip)	3049 ± 45	-25.2 ± 0.4	68,2% prob<br>1391 – 1336 BCE (29,1%)<br>1322 – 1258 BCE (34,4%)<br>1244 – 1233 BCE (4,7%)<br>95,4% prob<br>1422 – 1192 BC (94,3%)<br>1142 – 1131 BC (1,1%)

Table S3. GMM. The table shows the results of the LDA performed on the archaeological sample with different probability thresholds, above; the model error estimated on the basis of the allocation to domestic and wild groups of known modern specimens from the reference collection, in the middle part; the comparison between the model error and the proportion of domesticated pips in the archaeological sample, at the bottom.

**RESULTS ARCHEOLOGICAL SAMPLE**									
**Site**	**Chronology (Southern Italy)**	**Total n. of seeds**	**Threshold**	**Wild**	**Dom**	**NA**	**% wild**	**% dom**	**% NA**
**Grotta di Pertosa**	MBA 3 / LBA	55	p ≥ 0.5	44	11	NA	80,00%	20%	NA
			p ≥ 0.75	38	9	8	69,10%	16,40%	14,50%
			p ≥ 0.9	32	7	16	58,20%	12,70%	29,10%

**ERROR MODEL**								
**Grape subspecies**	**Total n. of seeds**	**Threshold**	**Wild**	**Dom**	**NA**	**% wild**	**% dom**	**% NA**
Vitis vinifera* subsp. *vinifera	2430	p ≥ 0.5	116	2314		4,77	95,22	
		p ≥ 0.75	78	2250	102	3,21	92,59	4,19
		p ≥ 0.9	49	2166	215	2,01	89,13	8,84
Vitis vinifera* subsp. *sylvestris	2430	p ≥ 0.5	2344	86		96,46	3,53	
		p ≥ 0.75	2268	37	125	93,33	1,52	5,14
		p ≥ 0.9	2170	14	246	89,3	0,57	10,12

**COMPARISON ERROR MODEL AND PROPORTION OF DOMESTICATED PIPS IN ARCHAEOLOGICAL SAMPLE**					
**p 0.5** : X-squared = 54.199, df = 2, p-value = 1.702e-12					
Group 1	Group 2	Nb	p	p.adj	p.adj.signif
REFSYL	Pertosa	2485	5,2E-06	1,57E-05	****
**p 0.75** : X-squared = 80.504, df = 2, p-value < 2.2e-16					
Group 1	Group 2	Nb	p	p.adj	p.adj.signif
REFSYL	Pertosa	2352	1,5E-07	4,35E-07	****
**p 0.90** : X-squared = 120.29, df = 2, p-value < 2.2e-16					
Group 1	Group 2	Nb	p	p.adj	p.adj.signif
REFSYL	Pertosa	2223	2,8E-08	8,52E-08	****

Table S4. Results of the LDA performed at cultivar level on the 9 pips identified as domesticated. Cases with p value over 0.75 were highlighted.

Pip ID	Cultivar	Probability	Sex	Color	Use	Region of origin
P2	Sliva	0,601726	H	Black	Table	Balkans
P50	Manizi	0,964559	nd	Red	Table	Balkans
P43	Bourboulenc	0,858817	H	White	Wine	West-Central Europe
P41	Genk Uzum	0,858809	F	White	Table	Caucaus & Near-East
P23	Kouldjinsky	0,692198	H	Pink	Dual	Balkans
P57	Assyrtico	0,657449	H	White	Wine	Balkans
P14	Chekobali	0,739716	H	White	Wine	Caucaus & Near-East
P17	Roussaïtis	0,897915	H	Red	Wine	Balkans
P36	Chekobali	0,399100	H	White	Wine	Caucaus & Near-East

SI References

1. Ramos-Madrigal, J. *et al.*, Palaeogenomic insights into the origins of French grapevine diversity. *Nature Plants* **5,** 595-603 10.1038/s41477-019-0437-5 (2019).
2. Knapp, M. Clarke, A.C. Horsburgh, K.A. & Matisoo-Smith, E.A. Setting the stage – Building and working in an ancient DNA laboratory. *Annals of Anatomy - Anatomischer Anzeiger* **194,** 3-6 10.1016/j.aanat.2011.03.008 (2012).
3. Wales, N. & Kistler, L. Extraction of Ancient DNA from Plant Remains in *Ancient DNA: Methods and Protocols* (eds. Shapiro, B. *et al.*) 45-55 (Springer, New York, NY, 2019), 10.1007/978-1-4939-9176-1_6
4. Dabney, J. *et al.* Complete mitochondrial genome sequence of a Middle Pleistocene cave bear reconstructed from ultrashort DNA fragments. *Proceedings of the National Academy of Sciences* **110,** 15758-15763 10.1073/pnas.1314445110 (2013).
5. Schrader, C. Schielke, A. Ellerbroek, L. & Johne, R. PCR inhibitors – occurrence, properties and removal. *Journal of Applied Microbiology* **113,** 1014-1026 10.1111/j.1365-2672.2012.05384.x (2012).
6. Sidstedt, M. Rådström, P. & Hedman, J. PCR inhibition in qPCR, dPCR and MPS—mechanisms and solutions. *Analytical and Bioanalytical Chemistry* **412,** 2009-2023 10.1007/s00216-020-02490-2 (2020).
7. Kapp, J.D. Green, R.E. & Shapiro, B. A Fast and Efficient Single-stranded Genomic Library Preparation Method Optimized for Ancient DNA. *J Hered* **112,** 241-249 10.1093/jhered/esab012 (2021).
8. Wales N. *et al.* New insights on single-stranded versus double-stranded DNA library preparation for ancient DNA. *BioTechniques* **59,** 368-371 10.2144/000114364 (2015).
9. Rohland, N. & Reich, D. Cost-effective, high-throughput DNA sequencing libraries for multiplexed target capture. *Genome Research* **22,** 939-946 10.1101/gr.128124.111 (2012).
10. Rohland, N. Harney, E. Mallick, S. Nordenfelt, S. & Reich, D. Partial uracil–DNA–glycosylase treatment for screening of ancient DNA. *Philosophical Transactions of the Royal Society B: Biological Sciences* **370,** 20130624 10.1098/rstb.2013.0624 (2015).
11. Laucou, V. *et al.* Extended diversity analysis of cultivated grapevine Vitis vinifera with 10K genome-wide SNPs. *PloS one* **13,** e0192540 10.1371/journal.pone.0192540 (2018).
12. Le Paslier, M-C. *et al.* A dataset of 9.896 single nuclear polymorphisms for 112 wild grapes, obtained with the GrapeReSeq 18K Vitis chip. Recherche Data Gouv. Available at https://doi.org/10.15454/9RUCEP. Deposited 19 April 2019.
13. Canaguier, A. *et al.* A new version of the grapevine reference genome assembly (12X.v2) and of its annotation (VCost.v3). *Genomics Data* **14,** 56-62 10.1016/j.gdata.2017.09.002 (2017).
14. Camacho, C. *et al.* BLAST+: architecture and applications. *BMC Bioinformatics* **10,** 421 10.1186/1471-2105-10-421 (2009).
15. Bouby, L. *et al.* Tracking the history of grapevine cultivation in Georgia by combining geometric morphometrics and ancient DNA. *Vegetation History and Archaeobotany* **30,** 63-76 10.1007/s00334-020-00803-0 (2020).
16. Schubert, M. *et al.* Characterization of ancient and modern genomes by SNP detection and phylogenomic and metagenomic analysis using PALEOMIX. *Nature Protocols* **9,** 1056-1082 10.1038/nprot.2014.063 (2014).
17. Jansen, R.K. *et al.* Phylogenetic analyses of Vitis (Vitaceae) based on complete chloroplast genome sequences: effects of taxon sampling and phylogenetic methods on resolving relationships among rosids. *BMC Evolutionary Biology* **6,** 32 10.1186/1471-2148-6-32 (2006).
18. Li, H. & Durbin, R. Fast and accurate short read alignment with Burrows–Wheeler transform. *Bioinformatics* **25,** 1754-1760 10.1093/bioinformatics/btp324 (2009).
19. McKenna A. *et al.* The Genome Analysis Toolkit: A MapReduce framework for analyzing next-generation DNA sequencing data. *Genome Research* **20,** 1297-1303 10.1101/gr.107524.110 (2010).

20. Jónsson, H. Ginolhac, A. Schubert, M. Johnson, P.L.F. & Orlando, L. mapDamage2.0: fast approximate Bayesian estimates of ancient DNA damage parameters. *Bioinformatics* **29,** 1682-1684 10.1093/bioinformatics/btt193 (2013).
21. Jun, G. Wing, M.K. Abecasis, G.R. & Kang, H.M. An efficient and scalable analysis framework for variant extraction and refinement from population-scale DNA sequence data. *Genome Research* **25,** 918-925 10.1101/gr.176552.114 (2015).
22. Danecek P. *et al.* Twelve years of SAMtools and BCFtools. *GigaScience* **10,** giab008 10.1093/gigascience/giab008 (2021).
23. R Core Team, R: A Language and Environment for Statistical Computing. R Foundation for Statistical Computing. https://www.R-project.org/ (2022).
24. Patterson, N. Price, A.L. & Reich, D. Population Structure and Eigenanalysis. *PLOS Genetics* **2,** e190 10.1371/journal.pgen.0020190 (2006).
25. Price, A.L. *et al.* Principal components analysis corrects for stratification in genome-wide association studies. *Nature Genetics* **38,** 904-909 10.1038/ng1847 (2006).
26. Wickham, H. *ggplot2: Elegant Graphics for Data Analysis* (Springer Publishing Company, Incorporated, 2009).
27. Chang, C.C. *et al.* Second-generation PLINK: rising to the challenge of larger and richer datasets. *GigaScience* **4,** s13742-13015-10047-13748 10.1186/s13742-015-0047-8 (2015).
28. Manichaikul, A. *et al.* Robust relationship inference in genome-wide association studies. *Bioinformatics* **26,** 2867-2873 10.1093/bioinformatics/btq559 (2010).
29. Korneliussen, T.S. Albrechtsen, A. & Nielsen, R. ANGSD: Analysis of Next Generation Sequencing Data. *BMC Bioinformatics* **15,** 356 10.1186/s12859-014-0356-4 (2014).
30. Hanghøj, K. Moltke, I. Andersen, P.A. Manica, A. & Korneliussen, T. S.Fast and accurate relatedness estimation from high-throughput sequencing data in the presence of inbreeding. *GigaScience* **8,** giz034 10.1093/gigascience/giz034 (2019).
31. Gansauge, M-T. & Meyer, M. Single-stranded DNA library preparation for the sequencing of ancient or damaged DNA. *Nature Protocols* **8,** 737-748 10.1038/nprot.2013.038 (2013).
32. Costantini, L. Costantini Biasini, L. La viticoltura dalla Grecia alla Magna Grecia: la documentazione archeobotanica in *Alle radici della civiltà del vino in Sicilia: atti del convegno Alle radici della civiltà del vino nel Mediterraneo: 3000 anni di storia (Menfi, 6 luglio 1996)*. (eds. Failla, O. Forni, G.) 169-192 (Cantine Settesoli, 1999).
33. Costantini, L. Italia centromeridionale in *Storia dell'Agricoltura Italiana, 1-2: L' età antica* (eds. Forni, G. & Marcone, A.) 221-234 (Accademia dei Georgofili, Firenze, 2002)
34. Delle Donne, M. La protostoria agricola della Campania: nuovi dati archeobotanici, *Rivista di Scienze Preistoriche* **LXVIII,** 305-358 (2018).
35. Costantini, L. Costantini Biasini, L. Delle Donne, M. L'agricoltura del villaggio preistorico di Nola, loc. Croce del Papa (NA) in *Atti della XL Riunione Scientifica dell'Istituto Italiano di Preistoria e Protostoria. Strategie di insediamento fra Lazio e Campania in età preistorica e protostorica* (Istituto Italiano di Preistoria e Protostoria, Firenze, 2007) 705-718.
36. Nilsson, M. Evidence of Palma Campania settlement at Pompeii in *Nuove ricerche archeologiche nell'area vesuviana (scavi 2003-2006)* (eds. Guidobaldi, M.P. & Guzzo P.G.) 81-86 (Studi della Soprintendenza Archeologica di Pompei, Pompei, 2008).
37. Nilsson, M. & Robinson, M. Remains of prehistoric habitation beneath Pompeii V 1, 13 *Opuscula Romana* **30,** 97-103 (2005).
38. Laforgia E. *et al.* The Vesuvian "Pomici di Avellino" eruption and Early Bronze Age settlement in the middle Clanis valley, *Mèditerranée*, **112,** 101-107 10.4000/mediterranee.3253 (2009).
39. Laforgia, E. Boenzi, G. Matarazzo, T. Stanzione, M. Ambiente e biodiversità nella piana campana: modalità di sfruttamento delle risorse vegetali in una comunità del Bronzo antico. Il villaggio del Bronzo antico di Afragola. *Studi di preistoria e protostoria* **6,** 275-283 (2021).
40. Caracuta, V. Fiorentino, G. & Martinelli, M.C. Plant Remains and AMS: Dating Climate Change in the Aeolian Islands (Northeastern Sicily) during the 2nd Millennium BC, *Radiocarbon* **54,** 3-4, 689-700 10.1017/S0033822200047354 (2012).

41. D’Oronzo, C. & Fiorentino, G. Archaeobotanical and spatial analysis of functional activities near hearth structures: the Bronze age settlements of Coppa Nevigata and Oratino (Italy), in *Actes des XXX Rencontres Internationales D’Archeologie et d’Histoire d’Antibes des hommes et des plantes: Exploitation du milieu et gestion des ressources végétales de la Préhistoire à nos jours* (eds. Delhon, C. Théry-Parisot, I. Thiébault, S.) 413-427 (Éditions APDCA, Antibes, 2010).
42. Martinelli, M.C. *et al.* Nuove ricerche nell'insediamento sull'istmo di Filo Braccio a Filicudi. Nota preliminare sugli scavi 2009, *Origini*, 32, 285-314 (2010).
43. Speciale, C. D'Oronzo, C. Stellati, A. & Fiorentino, G. Ubi Minor… deinde summa? Archaeobotanical data from the prehistoric village of Filo Braccio (Filicudi, Aeolian Archipelago): spatial analysis, crop production and paleoclimate reconstruction, *Scienze dell'Antichità,* **22,** 281-298 (2016).
44. Mangafa, M. & Kotsakis, K. A new method for the identification of wild and cultivated charred grape seeds. Journal of archaeological science 23, 3, 409-418 10.1006/jasc.1996.0036 (1996).
45. Fiorentino, G. Colaianni, G. Grasso, A.M. Stellati, A. Caratteristiche del paleoambiente e modalità di sfruttamento dei vegetali a Salina nel corso dell’Età del Bronzo in *Il villaggio dell'età del Bronzo di Portella nelle Isole Eolie (scavi 2006-2008)* 233-240 (ed. Martinelli, M.C.) (Rebus Edizioni, Milazzo, 2010).
46. Martinelli, M.C. *et al.* Nuove ricerche sul sito dell'età del Bronzo di Portella nell'isola di Salina in *Atti della XXXV Riunione Scientifica dell’Istituto Italiano di Preistoria e Protostoria, II* (Istituto Italiano di Preistoria e Protostoria, Firenze, 2003) 883-888.
47. Stummer, A. Zur Urgeschichte der Rebe und des Weinbaues. *Mitteilungen der Anthropologischen Gesellschaft in Wien* **41,** 283-296 (1911).
48. Perret, M. Polymorphisme des génotypes sauvages et cultivés de Vitis vinifera L. à l'aide de marqueurs RAPD. *Bulletin de la société Neuchâteloise des sciences naturelles* **120,** 45-54 (1997).
49. Fiorentino, G. & Agrostelli, M. Grotta della Madonna, Praia a Mare (Cosenza): archeocarpologia dei livelli olocenici (nuovi dati dagli scavi 2002-2004)" in *Preistoria e Protostoria della Calabria II. Scavi e Ricerche 2004-2005. Atti delle giornate di studio, Pellaro (RC), 22-23 ottobre 2005* (eds. Scarfò, M.S. Tinè, V.) 29-43 (Gruppo archeologico pellarese, Pellaro, 2007).
50. Peña-Chocarro, L. & Rottoli, M. La campagna di scavo 2002 nella Grotta della Madonna di Praia a Mare a (Cosenza). Appendice 2: le analisi archeobotaniche in *Atti della XXXVII Riunione Scientifica “Preistoria e protostoria della Calabria”, Scalea, Papasidero, Praia a Mare, Tortora, 29 settembre – 4 ottobre 2002* (Istituto Italiano di Preistoria e Protostoria, Firenze, 2004) 800-803.
51. D’Oronzo, C. & Fiorentino, G. Le analisi archeobotaniche nel sito dell’età del Bronzo di Oratino (Cb) Loc. La Rocca: implicazioni paleoeconomiche, paleoecologiche e modalità di funzionamento delle strutture pirotecniche in *Atti del 28° Convegno Nazionale sulla Preistoria, Protostoria e Storia della Daunia* (ed. Gravina, A.) 275-298 (San Severo, 2008).
52. D’Oronzo, C. & Fiorentino, G. Archaeobotanical and spatial analysis of functional activities near hearth structures: the Bronze age settlements of Coppa Nevigata and Oratino (Italy) in *Actes des XXX Rencontres Internationales D’Archeologie et d’Histoire d’Antibes des hommes et des plantes: Exploitation du milieu et gestion des ressources végétales de la Préhistoire à nos jours* (eds. Delhon, C. Théry-Parisot, I. Thiébault, S.) 413-427 (Éditions APDCA, Antibes, 2010).
53. Costantini, L. I resti vegetali carbonizzati di Vivara in *La ricerca archeologica a Vivara e le attività dei laboratori dell’Istituto Universitario Suor Orsola Benincasa* (ed. Pepe, C.) 83-87 (Napoli, 2001).
54. Ross Holloway, R. Buccino: the Early Bronze Age village of Tufariello, *Journal of Field Archaeology*, **2,** 1/2, 11-81 10.2307/529618 (1975).

55. Coubray, S. Étude anthracologique et carpologique in *Torre Mordillo 1987–1990. Le relazioni egee di una comunità protostorica della Sibaritide* (eds. Trucco, F. Vagnetti L.) 419-431 (Edizioni CNR, Roma, 2001).
56. Breglia, F. *Il sito palafitticolo ipogeo di Grotta di Pertosa (SA) nell'età del Bronzo: ricerche archeobotaniche*. Doctoral dissertation (University of Salento, 2020).
57. Barker, G. *A Mediterranean valley. Landscape archaeology and annales history in the Biferno Valley* (Bloomsbury Publishing PLC, London, 1995).
58. Jung, R. Pacciarelli, M. Zach, B. Klee, M. & Thanheiser, U. Punta di Zambrone (Calabria) a Bronze Age Harbour Site. First Preliminary Report on the Recent Bronze Age (2011-2012 Campaigns) *Archaeologia Austriaca*, **99/2015,** 53-110 10.1553/archaeologia99s53 (2015).
59. Primavera, M. *Roca e le dinamiche uomo-ambiente in Puglia durante l'età del Bronzo. Le piante e l'uomo tra ambiente paesaggio economia e culto* (edizioni Esperidi, Lecce, 2018).
60. Fiorentino, G. Caratteristiche della vegetazione e abitudini alimentari durante la preistoria in *Le Isole Chéradi fra natura, leggenda e storia.* (eds. Mastronuzzi, G. & Marzo, P.) 69-78 (Stampasud, 1999).
61. Nisbet, R. & Ventura, G. I dati archeobotanici in *Enotri e Micenei nella Sibaritide* (eds. Peroni, R. Trucco, F.) 577-585 (Istituto per la storia e l'archeologia della Magna Grecia, Taranto, 1994).
62. Peroni, R. Le comunità enotrie della Sibaritide ed i loro rapporti con i navigatori egei in *Enotri e Micenei nella Sibaritide* (eds. Peroni, R. Trucco, F.) 832-879 (Istituto per la storia e l'archeologia della Magna Grecia, Taranto, 1994).
63. Stika, H.P. Heiss, A.G. Zach, B. Plant remains from the early Iron Age in western Sicily: differences in subsistence strategies of Greek and Elymian sites, *Vegetation History and Archaeobotany*, **17,** 1,139-148 10.1007/s00334-008-0171-9 (2008).
